# Translation, cross-cultural adaptation to Brazilian Portuguese and measurement properties of the WaLIDD score

**DOI:** 10.61622/rbgo/2024AO16

**Published:** 2024-03-15

**Authors:** Mariana Arias Avila, Guilherme Tavares de Arruda, Amanda Garcia de Godoy, Patricia Driusso

**Affiliations:** 1 Universidade Federal de São Carlos São Carlos SP Brazil Universidade Federal de São Carlos, São Carlos, SP, Brazil.

**Keywords:** Women's health, Pain, Dysmenorrhea, Surveys and questionnaires

## Abstract

**Objective:**

Dysmenorrhea is the pain related to menstruation; to screen for the symptoms, a working ability, location, intensity of days of pain, and dysmenorrhea (WaLIDD) score was created. The purpose of this work was to culturally adapt and assess the measurement properties of the WaLIDD score for dysmenorrhea in Brazilian women.

**Methods:**

In this cross-sectional online study, we evaluated women with and without dysmenorrhea. Criterion validity and construct validity were assessed, respectively, by the Receiver Operator Characteristic (ROC) curve and correlations with the bodily pain and social functioning domains of medical outcomes study 36-item short-form health survey (SF-36), self-report of absenteeism and Stanford Presenteeism Scale for presenteeism. Test-retest reliability and measurement errors were assessed, respectively, by intraclass correlation coefficient (ICC) and Bland and Altman Graph.

**Results:**

430 women completed the test, 238 (55.4%) women had dysmenorrhea, and 199 (46.3%) answered the questionnaire twice for the retest. The cutoff points ≥4, ≥5, and ≥5 could discriminate between women with and without dysmenorrhea, absenteeism, and presenteeism related to dysmenorrhea, respectively. Correlations between SF-36 – pain and social functioning domains and WaLIDD score were weak to strong and negative. For WaLIDD total Score, ICC was 0.95 and the limits of agreement were −1.54 and 1.62.

**Conclusion:**

WaLIDD score is a short, valid and reliable instrument to screen and predict dysmenorrhea and could predict absenteeism and presenteeism related to dysmenorrhea in Brazilian women.

## Introduction

Dysmenorrhea is the pain related to menstruation that starts before or during the menses.^(1)^ The pain often occurs in the lower abdomen and/or lower back, but some women may experience pain in the head, legs, and breasts. In addition to pain, women may experience nausea, vomiting, fatigue, diarrhea, insomnia, emotional changes, and others.^(1,2)^ Thus, the pain experience can negatively impact the quality of life of the person with dysmenorrhea and the skills at work and/or school,^(3)^ directly affecting social relationships.^(2,4)^ A recent study showed that about 50% of women present central sensitivity symptoms, which may lead to central sensitization and thus to chronic pain syndromes such as fibromyalgia and migraine, and that those symptoms are more present in women with dysmenorrhea.^(5)^

Physiologically, dysmenorrhea can be classified as primary, when no pelvic disease is associated with pain, and secondary, when dysmenorrhea is associated with a pelvic disease, such as endometriosis, adenomyosis, uterine fibroid, and others.^(2)^ Although the prevalence of dysmenorrhea in young women worldwide is about 71%,^(3)^ it can be underestimated since many women do not consider it a health problem and do not seek professional help. Thus, self-reported tools to screen dysmenorrhea could help health professionals to diagnose^(6)^ and propose self-care strategies.

However, there are no validated Patient-Reported Outcome Measures (PROM) for Brazilian Portuguese for dysmenorrhea screening. The Working ability, Location, Intensity, Days of pain, Dysmenorrhea (WaLIDD) score was developed in Colombia to identify women with dysmenorrhea and those at high risk of sick leave.^(7)^ WaLIDD score seems to be a useful and rapid PROM for screening dysmenorrhea. This PROM has 14 items, but only 4 compose the total score. These items assess the number of anatomical pain locations, pain intensity, number of days of menstrual pain, and frequency of disabling pain to perform activities.^(7)^

In addition to the lack of specific PROMs for dysmenorrhea in Brazil, WaLIDD can to discriminate between women with and without dysmenorrhea, and is able to predict absenteeism related to dysmenorrhea. Thus, this PROM is useful in screening symptoms. Furthermore, WaLIDD score presents appropriate content validity (degree to which the content of a PROM is an adequate reflection of the construct to be measured), criterion validity (degree to which the scores of a PROM are an adequate reflection of a gold standard), internal consistency (degree of the interrelatedness among the items) and hypothesis test for construct validity (degree to which the scores of a PROM are consistent with hypotheses based on the assumption that the PROM validly measures the construct to be measured).^(8)^ However, the test-retest reliability (proportion of the total variance in the measurements which is due to ‘true’ differences between patients over time) and measurement errors (systematic and random error of a patient's score that is not attributed to true changes in the construct to be measured)^(8)^ were not evaluated. Thus, this study aimed culturally adapt and assess the measurement properties (criterion validity, hypothesis test for construct validity, test-retest reliability, and measurement errors) of the WaLIDD score for dysmenorrhea in a sample of Brazilian women.

## Methods

This cross-sectional online study was conducted between August 2020 and August 2021. The study was approved by the institutional Research Ethics Committee under the CAAE number 30232920.3.0000.5504. After reading the Informed Consent Term, all participants were granted consent by clicking on the button "I declare that I have read the above term, understood it, and agree to participate in the research". We followed the recommendations of the Consensus-based Standards for the Selection of Health Measurement Instruments (COSMIN)^(9)^ for assessing the measurement properties of PROM.

All participants were recruited through Facebook, Instagram, and WhatsApp. Women aged over 18 years, literate, and with access to the Internet were included. We excluded postmenopausal, pregnant, and postpartum women up to twelve months after delivery, women with any pelvic infection (urinary tract infection, sexually transmitted, and others), and who had not menstruated in the last three months.

Participants answered an initial anamnesis on Google Forms and were contacted by a researcher close to the expected next menstruation date. In the anamnesis, the participants were asked about age, if they were pregnant, the date of the last delivery and last menstruation, if they had any pelvic infection, and contact for the return of the questionnaire. Participants answered the WaLIDD score between the 6th and 9th day of the menstrual cycle. All participants were contacted for the second assessment 7 days later for retest analysis. We got participants via text messages and all questionnaires were self-administered.

For sample size, we relied on COSMIN's sample size recommendation for validation studies – five to seven times the number of items in the instrument and greater than 100 participants.^(9)^ Considering the number of WaLIDD score items and that participants with and without dysmenorrhea would be included, we expected a minimum of 200 participants.

Translation and content validity occurred in 5 steps,^(10)^ after the WaLIDD's developers authorized the translation and validation into Portuguese-Brazil. In the first step, the WaLIDD score was translated by two independent translators fluent in Spanish, whose native language is Portuguese-Brazil (T1 and T2). In the second step, the discrepancies in translations were discussed and synthesized in consensus (T12) by two translators and researchers. In the third step, this version (T12) was sent to two back translators whose native language is Spanish. Both back translators did not know the original version of WaLIDD score and did not have specific knowledge of the health area for back-translation (B1 and B2). In the fourth step, the four versions (T1, T2, B1 and B2) were synthesized and evaluated by an expert committee composed of a methodologist, two physical therapists, a letterologist and translators for the assessment of semantic, language, experimental and conceptual equivalences, generating a pre-final version. In the fifth step, the pre-final version was tested with adult women from different educational levels, who responded to a semi-structured interview, online or by telephone, containing questions about the questionnaire's clarity and any suggestions that participants thought were needed to improve the questionnaire.

Initially, we assessed the sociodemographic and menstrual characteristics with a questionnaire developed by the authors. For the assessment of dysmenorrhea self-report, the following question was considered: "Do you have menstrual cramps?". This question was assessed in the initial anamnesis and the answer options were "yes" or "no". Along with that, women should answer their self-perception of their menstrual pain intensity using the Numerical Rating Scale (NRS). This instrument was already used to assess women with dysmenorrhea, with ICC = 0.90, and an area under the receiver operating characteristic of 0.902 (95%CI, 0.873–0.931) and a cutoff score of 3 points.^(11)^ We considered women with dysmenorrhea if their pain was rated >3 on the NRS.

The WaLIDD score is self-administered to discriminate between women with and without dysmenorrhea and predict high risk of sick leave. This instrument has 14 items, in which 4 items assess pain locations, pain range, number of days of menstrual pain and frequency of disabling pain to perform activities for the total score, and 10 other items about anamnesis, on pain and drug use assessed by a verbal scale. The 10 items do not count in the total score. The 4 items in total score are scored from zero (no dysmenorrhea) to 3 (presence in the most severe pain). For drug score, it is assessed the number of days of analgesic use during menstruation from zero (Do not use) to 3 (3 days).^(7)^

In the first assessment, the participants answered the bodily pain and social functioning domains of medical outcomes study 36-item short-form health survey (SF-36), self-report of absenteeism and Stanford Presenteeism Scale (SPS-6) for presenteeism. The Medical outcomes study 36-item short-form health survey (SF-36) assesses negative and positive health aspects on eight scales: functional capacity, physical aspects, pain, general health, vitality, social aspects, emotional aspects and mental health, in addition to comparing the current health with that of a year ago. It has been validated in Portuguese, with moderate to good intra- and inter-rater reliability (0.44 < r <0.85 and 0.55 < r < 0.81). We use SF-36 pain scale to assess the frequency or discomfort of pain and its interference with normal activities, and functional capacity to assess health-related effects on social activities. Lower scores in these domains indicate worse quality of life.^(12)^

To assess self-reported absenteeism, we used the following question: "In the last three months, did you miss a day of work/class due to menstrual cramps?". The response options were "yes" or "no". Presenteeism was assessed by SPS-6 in the last 30 days. The SPS-6 has 6 items with 5 response options (strongly disagree to strongly agree), in which the higher the score, the worse the presenteeism. This two-factor scale presented adequate values for test-retest reliability (ICC=0.91) and internal consistency (α>0.70) in the validation in Brazilian Portuguese.^(13)^

The 15-points Global Rating of Change Scale (GRCS)^(14)^ was used to differentiate participants with changes in dysmenorrhea complaints between test and retest. We asked participants "Regarding your period pain, how would you describe it now compared to when it started?" with answers from "a very great deal better" to "a very great deal worse". We considered variations up to 2 points in the GRCS to classify patients who did not change their dysmenorrhea complaints between test and retest.

The results were expressed in percentages or mean and standard deviation (SD). The Kolmogorov-Smirnov test was used for analysis of normality, and the Mann-Whitney test for quantitative variables, Chi-square and Fisher's exact test for categorical variables, in the analysis of sample characterization. A significance level of 5% was considered in the analyses. This analysis, hypothesis test for construct validity, test-retest reliability and measurement errors were performed in SPSS 22.0. Criterion validity was assessed in MedCalc 20.011.

Criterion validity was assessed by AUC-ROC, sensitivity and specificity for dysmenorrhea, presenteeism and absenteeism. For dysmenorrhea analysis, we considered the WaLIDD total score and dysmenorrhea self-report. Considering the impact of dysmenorrhea on presenteeism, we analyze WaLIDD total score and SPS-6 total score as indicative of presenteeism (between 6 and 18) or not (above 18).^(13)^ For absenteeism, we considered the WaLIDD total score and the self-report of medical leave due to menstrual cramps in the last three months. The area under the ROC curve ≥0.8 was considered to indicate excellent accuracy.

To assess the hypothesis test for construct validity, we used Spearman's correlation according to the following Cohen^(15)^ correlation magnitudes: weak (0.10>rho<0.30), moderate (0.30>rho<0.50) and strong (rho≥0.50). We expected: (1) moderate to strong negative correlations between SF-36 pain domain and items 5 (days of pain), 7 (pain locations) and total score of WaLIDD; (2) moderate to strong negative correlations between SF-36 social functioning domain and item 8 (frequency of disabling pain to perform activities) of WaLIDD; and (3) weak to moderate negative correlation between SF-36 pain domain and item 6 (pain range) of WaLIDD. To verify the difference in the WaLIDD total score between the groups of women with and without dysmenorrhea, we used the Mann-Whitney U test and the hypothesis that there is a significant difference (p<0.05) between the groups for the WaLIDD total score.

Test-retest reliability and measurement errors were assessed for women who did not modify complaints of dysmenorrhea by the GRCS between 7 to 10 days. We use the intraclass correlation coefficient (ICC) with a two-way mixed-effect model with interaction for absolute agreement between mean measures. ICC > 0.75 was considered acceptable reliability.^(16)^ For measurement errors, we calculated the Standard Error of the Measurement (SEM), Smallest Detectable Change (SDC) at the individual level and the Bland and Altman graph. SEM was calculated by the SD of the difference between the test and retest WaLIDD total score, divided by √2 (17). SDC was calculated as SEM × 1.96 × √2.^(17)^ Bland and Altman graph was analyzed by the limits of agreement (LoA) using the formula [d- ± (1.96 × diferenceSD)], where d- is the mean of the differences the test and retest WaLIDD scores, and diferenceSD is SD of the mean of the differences the test and retest WaLIDD scores.^(17)^

This study was performed in line with the principles of the Declaration of Helsinki. Approval was granted by the Ethics Committee of Universidade Federal de São Carlos (UFSCar) (CAAE 30232920.3.0000.5504, approval date 23/05/2020).

## Results

Six hundred and thirty-eight women responded to the initial anamnesis. Of these, 125 (19.59%) were not included due to contact difficulties, such as incorrect or unanswered telephone and email numbers, 92 (14.42%) according to the exclusion criteria; thus, 430 women completed the test and 199 (46.28%) completed the retest. The characteristics of the study participants are shown in table 1. According to the self-report of dysmenorrhea, 238 (55.35%) women had dysmenorrhea and 192 (44.65%) women did not report dysmenorrhea. There was a significant difference between women with and without dysmenorrhea in the menstrual cycle length (p = 0.040), menstrual flow length (p = 0.001), and use of pain medication for dysmenorrhea (p < 0.001).

**Table 1:** Characteristics of the study participants

Characteristics		Women with dysmenorrhea (n=238) n(%)	Women without dysmenorrhea (n=192) n(%)	p-value
Age (years), mean ± SD		25.96 ± 0.42	25.47 ± 0.36	0.41
Marital status, n (%)				0.93
	In a relationship	67(28.15)	40(20.83)	
	Not in a relationship	171(71.85)	152(79.17)	
Educational level, n (%)				0.74
	Primary education	3(0.26)	0(0)	
	Secondary education	27(11.34)	13(6.77)	
	Tertiary education	208(87.39)	179(93.23)	
Age of menarche, mean ± SD		11.94 ± 0.09	12.15 ± 0.10	0.15
Menstrual cycle length, mean ± SD		28.26 ± 3.99	28.71 ± 3.42	0.040*
Menstrual flow length, mean ± SD		2.13 ± 0.57	2.31 ± 0.55	0.001*
Use of pain medication for dysmenorrhea, n (%)				<0.001*
	Yes	198(83.19)	144(75)	
	No	40(16.81)	48(25)	
Pregnancies				0.91
	Nulliparous	197(82.77)	156(81.25)	
	Primiparous	22(9.24)	20(10.42)	
	Multiparous	19(7.98)	16(8.33)	
Polycystic Ovarian Syndrome, n (%)				0.591
	Yes	10(4.20)	8(4.17)	
	No	228(95.80)	184(95.83)	

SD - standard deviation;

* p<0.05

**Figure 1:**
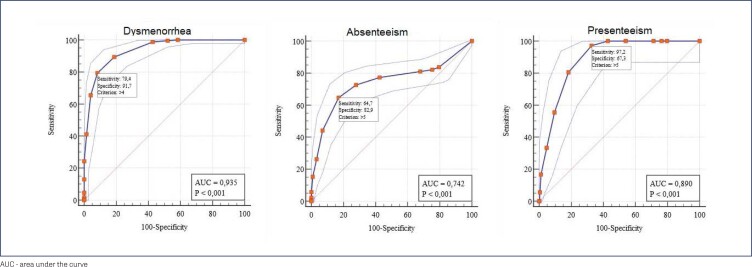
Criterion validity of the Brazilian version of WaLIDD score

Regarding the translation and content validity of WaLIDD score, the main adaptation was the use of an online form rather than the original paper and the addition of a definition of recall time of 3 months to questions 5 to 8. Thirty-five women (19 and 45 years old) participated in fifth step of content validity. The instrument was generally well understood by women. Women suggested adding the definition of the menstrual cycle and the use of upper-case letters for the recall time to ensure that it was taken under consideration. For item 5 (days of pain), we changed the modified Wong-Baker scale for an adaptation, designed by our group, similar to the Faces Pain Scale – Revised (FPS-R) (Hicks et al., 2001)^(18)^ because the FPS-R has a neutral non-pain face as pain intensity. The Brazilian version of WaLIDD score is presented in appendix a. For criterion validity, 430 women (238 with dysmenorrhea and 192 without dysmenorrhea) were analyzed. The cutoff point ≥4 of WaLIDD was able to discriminate between women with and without dysmenorrhea (AUC = 0.94, sensitivity = 79.4% and specificity = 91.7%). To discriminate absenteeism (AUC = 0.89, sensitivity = 97.2% and specificity = 67.3%) and presenteeism (AUC = 0.74, sensitivity = 64.7% and specificity = 82.9%), both had a cutoff point ≥5 (Figure 1).

In table 2, the correlations between the items, total score of the WaLIDD and the pain and social functioning domains of the SF-36 are presented. Correlations between SF-36 - pain domain and items 5 (days of pain), 6 (pain range), 7 (pain locations) and total score of WaLIDD were moderate to strong negative (rho = −0.47 to −0.57). For item 8 (perform activities), SF-36 - social functioning domain presented a weak negative correlation (rho = −0.25). When comparing the WaLIDD total score between groups of women with (n = 238) and without dysmenorrhea (n = 192), the mean and SD of the scores for both groups were, respectively, 6.54 ± 1.87 and 2.41 ± 2.17 points, and there was a significant difference (p<0.001).

**Table 2:** Hypothesis testing for construct validity of WaLIDD score

WaLIDD item	Instrument	Rho	p-value
Item 5 - days of pain	SF-36 - pain domain	−0.51	<0.001*
Item 6 - pain range	SF-36 - pain domain	−0.47	<0.001*
Item 7 - pain locations	SF-36 - pain domain	-0.50	<0.001*
Item 8 - perform activities	SF-36 - social functioning domain	-0.25	<0.001*
WaLIDD total Score	SF-36 - pain domain	-0.57	<0.001*

SF-36 - Medical outcomes study 36-item short-form health survey; Rho - Spearman's correlation coefficient; WaLIDD - Working ability, Location, Intensity, Days of pain, Dysmenorrhea score.

* p<0.05

Among the 199 women in the retest, 152 (76.38%) self-reported dysmenorrhea. However, 105 (69,08%) women did not change dysmenorrhea complaints between test and retest by GRCS. For each WaLIDD item, the ICC ranged from 0.85 to 0.95. For the total score of WaLIDD, the ICC was 0.97 (Table 3).

**Table 3:** Test-retest reliability of WaLIDD score items and total score

WaLIDD item	ICC	95%CI
Item 5 - days of pain	0.90	0.85 - 0.93
Item 6 - pain range	0.93	0.89 - 0.95
Item 7 - pain locations	0.85	0.78 - 0.90
Item 8 - perform activities	0.92	0.88 - 0.95
WaLIDD total Score	0.95	0.92 - 0.96

ICC: Intraclass Correlation Coefficient. WaLIDD: Working ability, Location, Intensity, Days of pain, Dysmenorrhea score

**Figure 2:**
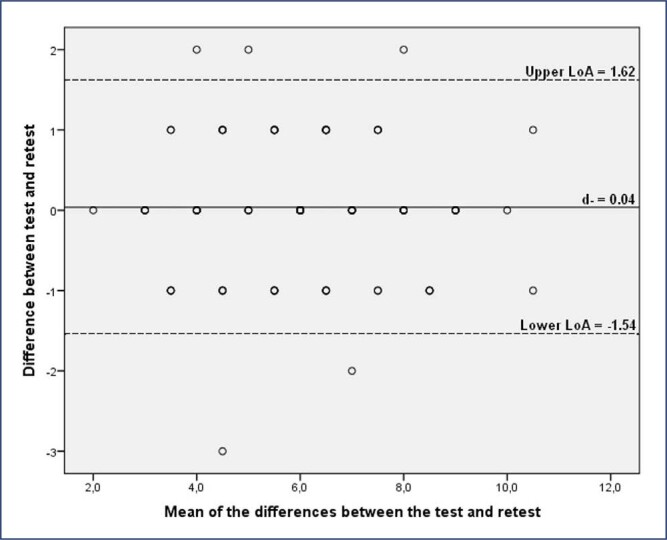
Bland Altman plot for the WaLIDD total score

For the total WaLIDD score, the mean difference (d-) between the test and retest was 0.04 point. There was no significant systematic bias between the WaLIDD test-retest measures because the 95%CI for the d- included zero (−0.12 to 0.19). SEM was 0.57 point and SDC at the individual level was 1.58 points. The LoA were −1.54 and 1.62, according to Bland & Altman graph presented in figure 2.

## Discussion

This study culturally adapted and assessed the measurement properties (criterion validity, hypothesis test for construct validity, test-retest reliability and measurement errors) of the WaLIDD score for dysmenorrhea in Brazilian women. In the translation and adaptation steps, we proposed changing the face scale used in the original WaLIDD to assess pain intensity. We also considered it important to include the recall time.^(19)^ Since the development of the WaLIDD score was in Colombia and there is no other PROM to assess dysmenorrhea in Brazil, the present study was the first to culturally adapt and assess the measurement properties of this instrument.

We present three cut-off points for the WaLIDD score about self-reported dysmenorrhea, absenteeism, and presenteeism. Thus, clinicians and researchers can discriminate between women who have and do not have dysmenorrhea, absenteeism and presenteeism from school and work in a single validated instrument. In the Colombian version of WaLIDD scoring, the authors observed a cut-off point of 6 to discriminate students with and without dysmenorrhea (AUC = 0.817).^(7)^ However, in the present study, the best cutoff point to discriminate dysmenorrhea was 4 points (AUC = 0.935). This difference between the cut-off points may have occurred because the discriminate variable, in Spanish version was used medication to relieve dysmenorrhea and, in our study, we used self-report (yes or no) as a criterion for dysmenorrhea. Thus, some women may experience dysmenorrhea, not using analgesics, but other non-pharmacological methods such as hot water bag, acupuncture, and physical therapy.^(20)^

The challenge on validating this instrument relates to the WaLIDD scoring: it is a screening instrument for dysmenorrhea with a formative model, in which the items do not necessarily correlate with each other.^(8)^ Thus, it makes no sense to assess the structural validity (degree to which the scores of an instrument are an adequate reflection of the dimensionality of the construct to be measured) and internal consistency of the WaLIDD score. For the evaluation of the hypothesis test for construct validity, the developers of the WaLIDD score used a verbal pain scale,^(7)^ but we used the SF-36 body pain domain. For this, we consider pain assessment as a complex construct that goes beyond intensity, and the SF-36 assesses pain related to quality of life.^(12)^ As expected, we found strong correlations between WaLIDD and SF-36 body pain domain. Thus, both instruments assess pain intensity and how it affects daily activities.^(7,12)^ In addition, there was a weak correlation between SF-36 functional capacity domain and WaLIDD's item 8. Although these measures may measure similar constructs, the activities assessed are discrepant. While WaLIDD assess interference at daily activities, such as work and studies, the SF-36 specifies simple physical actions, such as climbing stairs and walking small distances, that may be performed by women suffering from dysmenorrhea. Also, as we expected, we also found a significant difference between the groups of women with and without dysmenorrhea for the WaLIDD total score. Through this result, it is possible to state that the WaLIDD score can differentiate women with and without dysmenorrhea.

The values of test-retest reliability of the Brazilian version of WaLIDD score were excellent. This means that the WaLIDD total score is consistent to assess dysmenorrhea over time. In addition, when the ICC is inadequate, the dysmenorrhea screening would vary over time and could not be indicated in clinical practice.^(17)^ Our study was also the first to calculate measurement errors for the WaLIDD score. The measurement error of the WaLIDD score was 0.04 point and the minimum change necessary to make sure that a real change in the instrument occurred was 0.57 point. Also, Bland & Altman graph allowed visual assessment of how well the two measures agree. In this sample, there is no systematic bias, which it means that the WaLIDD score is an adequate measure to be used by healthcare professionals to assess dysmenorrhea.

In this study, we followed the COSMIN guidelines^(9)^ to assess the measurement properties of the WaLIDD score. In addition, this is the first Brazilian study to assess the measurement properties of an instrument to screen dysmenorrhea, demonstrating the need for its cultural adaptation and validation in this country. However, we had some limitations. more than 80% of participants with and without self-reported dysmenorrhea had tertiary education and had access to the internet. Thus, we suggest that future studies assess the measurement properties of WaLIDD for adolescents with this population.

## Conclusion

The WaLIDD score was translated and culturally adapted, and is a short, valid and reliable instrument to screen dysmenorrhea and also predict absenteeism and presenteeism in Brazilian women. Now clinicians and researchers can use this online instrument to screen, using the proposed cutoff scores of ≥4 to screen for dysmenorrhea and ≥5 to screen for absenteeism and presenteeism in Brazil.

## Appendix A WaLIDD Brazilian Version

**WaLIDD – Versão Brasileira**

**1. Qual é o seu nome completo? ___________________________________________________**

**2. Idade: ____________**

**3. Idade da sua primeira menstruação: ____________**

**4. Normalmente, qual a duração do seu ciclo menstrual?**

*Entende-se por ciclo menstrual o período entre uma menstruação e outra, ou seja, o primeiro dia da menstruação até o próximo primeiro dia da menstruação do mês seguinte.*

**Table:**

	< 21 dias
	28 a 30 dias
	35 dias
	Outro: ______________

**5. Qual a duração aproximada da sua menstruação, em dias? ___________________**

**6. Estas faces mostram o quanto algo pode doer. Esta face mais à esquerda indica não dor. As faces mostram cada vez mais dor até chegar a esta face mais à direita, que mostra muita dor. Marque a face que representa como você classifica a intensidade da sua dor menstrual NOS ÚLTIMOS TRÊS MESES:**

**7. Qual foi a localização mais frequente da sua dor menstrual, NOS ÚLTIMOS TRÊS MESES? *(Pode assinalar mais de uma opção)***

**8. Tendo em vista a duração do seu período menstrual, quantos dias sentiu dor durante o seu ciclo, NOS ÚLTIMOS TRÊS MESES?**

**Table:**

	Nenhum dia
	1 a 2 dias do ciclo
	3 a 4 dias do ciclo
	5 ou mais dias do ciclo

**9. A dor relacionada à sua menstruação lhe impossibilitou e/ou incapacitou frequentemente para realizar suas atividades diárias, NOS ÚLTIMOS TRÊS MESES?**

**Table:**

	Sempre
	Quase sempre
	Quase nunca
	Nunca

**10. Especifique as estratégias que utiliza para o controle da dor menstrual *(Pode assinalar mais de uma opção)***

**Table:**

	Descansar usando calor local (ex.: bolsa de água quente, compressas quentes)
	Tomar chás
	Tomar analgésicos convencionais e anti-inflamatórios
	Tomar anticoncepcionais orais
	Usar estímulo de pontos de acupuntura
	Usar "choquinho": estimulação nervosa transcutânea
	Praticar ioga, musicoterapia, aromaterapia

**11. Nos últimos 3 meses, foi incapacitada por causa da dor relacionada à menstruação?**

**Table:**

	Sim
	Não

**12. Se você toma analgésicos (remédios para a dor) durante a menstruação, por quantos dias os usa?**

**Table:**

	1 dia
	2 dias
	3 dias
	Não tomo

**13. Você foi diagnosticada com algumas das seguintes doenças ginecológicas? *(Pode assinalar mais de uma opção)***

**Table:**

	Endometriose
	Endometrite
	Tumor de origem ginecológica
	Miomatose uterina
	Salpingite
	Síndrome do ovário policístico
	Nenhuma das anteriores
